# Associations of Boiled Water and Lifespan Water Sources With Mortality: A Cohort Study of 33,467 Older Adults

**DOI:** 10.3389/fpubh.2022.921738

**Published:** 2022-06-27

**Authors:** Xun Liu, Zheng Pei, Zifan Zhang, Yan Zhang, Yongjie Chen

**Affiliations:** ^1^Department of Ultrasonics, Tianjin Fifth Central Hospital, Tianjin, China; ^2^Dean's Office, Tianjin Fifth Central Hospital, Tianjin, China; ^3^Department of Nutrition, Tianjin Fifth Central Hospital, Tianjin, China; ^4^Department of Epidemiology and Statistics, School of Public Health, Tianjin Medical University, Tianjin, China

**Keywords:** groundwater, surface water, tap water, boiled water, mortality

## Abstract

**Background:**

There were few studies to report whether drinking water sources and habits affected health outcomes. Therefore, this study aimed to examine how boiled water and lifespan water sources affected the risks of cardiovascular disease (CVD) and all-cause mortality in the elderly.

**Methods:**

This study was a 20-year cohort study. All participants aged ≥60 years were eligible. Exposures of interest included lifespan drinking water sources and habits, which were collected using a validated questionnaire. Drinking water sources included wells, surface water, spring, and tap water in childhood, around the age of 60 years, and at present. Drinking habits included boiled and un-boiled water. The main end events included CVD and all-cause mortality.

**Results:**

There were 33,467 participants in this study. Compared to tap water, drinking well and surface water around the age of 60 years were associated with a higher risk of all-cause mortality (*HR*: 1.092, *95% CI*: 1.051–1.134, *P* < 0.001; and *HR*: 1.136, *95% CI*: 1.081–1.194, *P* < 0.001, respectively). However, only drinking spring around aged 60 years and drinking well at present were associated with a lower CVD mortality (*HR*: 0.651, *95% CI*: 0.452–0.939, *P* = 0.022; and *HR*: 0.757, *95% CI*: 0.665–0.863, *P* < 0.001, respectively). Boiled water was not associated with mortality.

**Conclusions:**

Drinking water from well and surface water around the age of 60 years were associated with increased all-cause mortality. Drinking water from spring around the age of 60 years and well at present was associated with a decreased CVD mortality. However, boiled water was not associated with mortality.

## Introduction

As drinking water is an essential issue for human beings, access to safe drinking water is the most fundamental requirement for public health. It is reported that about 2.2 billion people have no access to improved drinking water worldwide (1). It is estimated that 785 million people suffer from the drinking water crisis, especially in rural areas of low- and middle-income countries (2). Contaminated water is still a major cause of infectious diarrheal diseases. As a result, there were approximately 500 thousand deaths attributed to inadequate safe water, which accounted for one-third of diarrhea-attributed deaths in 2016 (3). Therefore, access to sustainable and safe drinking water is still a major goal and global challenge of public health.

The associations of water, sanitation, and hygiene with health outcomes are critical for the prevention of diseases and improvement of life expectancy. Until now, there were many studies to report the associations of drinking water with health outcomes, including mortality and cardiovascular disease (CVD) (4–7). However, these studies are limited and have not investigated the associations of disinfection byproducts, compositions, hardness, and temperature of drinking water with health outcomes (7–11). In fact, investigating how drinking water sources and habits affect health outcomes is more significant from the perspective of public health. Furthermore, the quality of drinking water has been considerably improved due to the efforts of governments. For example, a large amount of capital has been spent to improve drinking water treatment and supply infrastructure in the last few decades in China (12). The main source of drinking water has changed from unimproved water, such as wells, rivers, and lakes, to improved water. Therefore, investigating the impact of lifespan drinking water sources and habits on health outcomes is particularly important, especially for frail populations, such as children and the elderly.

In this study, the Chinese Longitudinal Healthy Longevity Survey (CLHLS) was used to examine how boiled water and lifespan water sources affect CVD and all-cause mortality. As a result, this study would contribute to the improvement of health in the elderly.

## Materials and Methods

### Study Design and Population

The CLHLS was first conducted in 1998, and followed by waves in 2000, 2002, 2005, 2008–2009, 2011–2012, 2014, and 2017–2018. Twenty-three provinces/ municipalities were randomly selected from 31 provinces/ municipalities in China. Then, about half of the counties and city districts were randomly sampled within 23 provinces/ municipalities. In a healthy longevity survey, there were 19.5 thousand centenarians, 26.8 thousand nonagenarians, and 29.7 thousand octogenarians. The older people aged 80 years or older accounted for 67.4% of the total sample. A validated questionnaire was used to collect information on family structure, physical performance, health status, history of chronic diseases, and so on.

**Figure 1:**
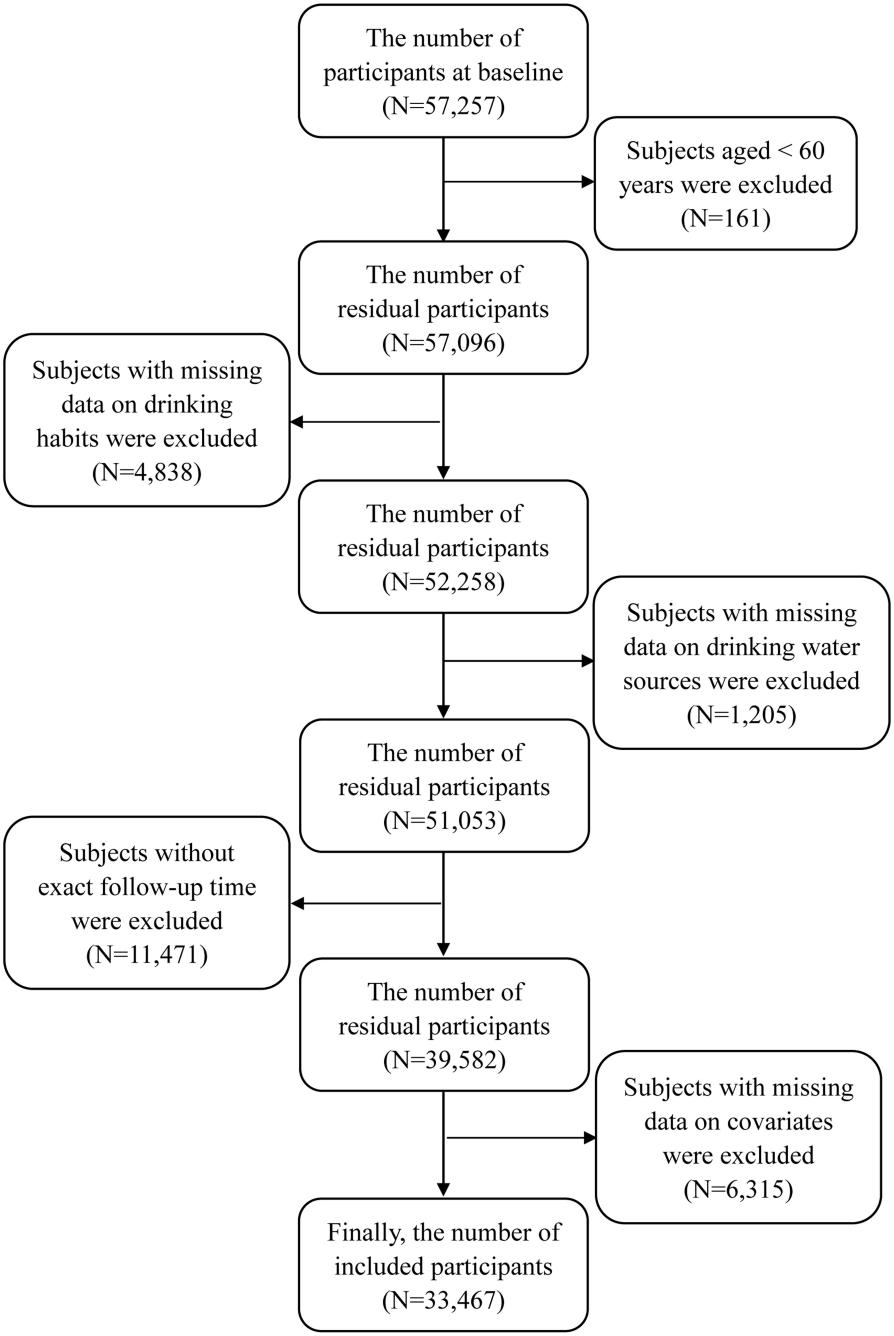
The process of participant selection in this study.

This study included all participants aged 60 years or older. If subjects had incomplete data on drinking water sources, drinking habits, and other covariates, they would be excluded. Meanwhile, subjects with abnormal data on covariates, such as weight and blood pressure, were also excluded. The detailed screening procedure is shown in Figure 1. This study was approved by the Institutional Review Board, Duke University (Pro00062871), and the Biomedical Ethics Committee, Peking University (IRB00001052–13074). All participants provided written informed consent.

### Measures

Exposures of interest included drinking water sources and drinking habits. Drinking water sources in childhood, around the age of 60 years, and at present were identified *via* a similar question: Water you drink is mainly from? Participants were required to select an answer from the following options: 1. well, 2. rivers or lakes, 3. spring, 4. pond or pool, and 5. tap water. In the final analysis, drinking water sources were classified as follows: tap water, well, surface water, and spring. Similarly, a question “What kind of water you usually drink?” was used to identify drinking habits, including boiled and un-boiled water.

### Death Assessment

End-events of interest included CVD mortality and all-cause mortality. In each wave, survival status was checked for each participant in a face-to-face interview. If someone died before a certain survey, the ascertainable date of death was identified by interviewing a close family member or local doctors. Furthermore, cause-specific mortality was further interviewed. Deceased participants caused by CVD were considered to have an end-event of CVD mortality.

### Covariates

In this study, information on age, sex, and ethnicity was collected according to participants' identification cards. Education levels and living areas were measured by self-report. Weight was measured by the study staff according to the standardized protocol. Dietary intakes at present were collected *via* the following questions: How often do you eat fresh fruit at present? How often do you eat vegetables at present? How often do you eat meat at present? How often do you eat fish at present? Information on alcohol consumption, current smoking habits, physical activity, self-rated health, and history of hypertension, diabetes, and CVD were collected using a validated questionnaire, which was described in detail elsewhere (13).

### Statistical Analysis

A *Kolmogorov-Smirnov test* was used to test for the normality of continuous variables. Continuous data with normal distribution, such as age and weight, were expressed as means ± standard deviations and were compared with the differences between-group using a *t-*test. Categorized variables were presented as frequencies (constituent ratios) and were compared with the differences between-group using the *chi-square* test. *Hazard ratios* (*HRs*) and *95% CIs* of drinking water sources and habits on the risks of CVD and all-cause mortality were estimated using *Cox* regressions with adjustments for age, sex, weight, ethnicity, education levels, marital status, living areas, current smoker, current alcohol consumer, physical activity, dietary intakes including fruits, vegetables, meat, and fish, history of hypertension, history of diabetes, self-reported health, and waves. Based on the adjusted factors mentioned above, drinking water source was additionally adjusted when drinking habits as the main exposure. Meanwhile, drinking habits were further adjusted based on the common- adjusted factors mentioned above when drinking water source as the main exposure. The Fine-Gray competing risk model was used to correct the competitive risk of death caused by other causes when CVD mortality is the end event. *Cox* regressions were further stratified by sex and living areas. In this study, the proportional hazards assumption was confirmed to hold in all *Cox* regressions. To prevent the possibility that deaths were caused by other fatal illnesses, sensitivity analysis was conducted with participants who died within the first 2 years after baseline being excluded from the analysis. All analyses were conducted using SAS 9.4 (SAS Institute Inc., Cary, NC, USA.). A two-tailed *P* ≤ 0.05 indicated a statistical significance.

## Results

### Characteristics of All Participants

Finally, there were 33,467 participants included in this study. The median time of follow-up was 5 years. The averages of age and weight were 86.38 ± 11.53 years and 49.60 ± 11.18 kg, respectively. The proportions of males and females were 43.33 and 56.67%, respectively. There were 31,904 and 1,563 participants drinking boiled and un-boiled water, respectively. There were 20,295 and 1,210 deceased participants caused by all-cause mortality and CVD, accounting for 60.64 and 3.80%, respectively. There were significant differences between censoring and end-event caused by all-cause mortality in all baseline characteristics, except a history of hypertension (*P* = 0.480). Meanwhile, significant differences between censoring and end-event caused by CVD were observed in all baseline characteristics, except weight (*P* = 0.141), marital status (*P* = 0.224), education (*P* = 0.065), ethnicity (*P* = 0.425), current smoking habits (*P* = 0.194), current alcohol consumption (*P* = 0.613), self-reported health (*P* = 0.238), history of diabetes (*P* = 0.905), and drinking habits (*P* = 0.185) as shown in Table 1.

**Table 1:** The characteristics of all participants at baseline.

**Characteristics**	**Total sample**	**All-cause mortality**			**CVD mortality**		
	**(*N =* 33,467)**						
		**Censoring**	**End-event**	***P***	**Censoring**	**End-event**	***P***
		**(*N =* 13,172)**	**(*N =* 20,295)**		**(*N =* 30,592)**	**(*N =* 1,210)**	
Age (years)^a^	86.38 ± 11.53	81.24 ± 11.98	89.71 ± 9.89	<0.001	86.38 ± 11.56	87.40 ± 9.53	<0.001
Weight (kg)^a^	49.60 ± 11.18	52.33 ± 11.67	47.83 ± 10.47	<0.001	49.50 ± 11.11	49.97 ± 10.90	0.141
**Sex** ^***b***^				<0.001			<0.001
Male	14,501 (43.33)	5,909 (44.86)	8,592 (42.34)		13,174 (43.06)	584 (48.26)	
Female	18,966 (56.67)	7,263 (55.14)	11,703 (57.66)		17,418 (56.94)	626 (51.74)	
**Marital status** ^** *b* **^				<0.001			0.224
Married	10,776 (32.20)	5,861 (44.50)	4,915 (24.22)		9,764 (31.92)	353 (29.17)	
Divorced	160 (0.48)	60 (0.46)	100 (0.49)		151 (0.49)	5 (0.41)	
Widowed	22,159 (66.21)	7,106 (53.95)	15,053 (74.17)		20,334 (66.47)	839 (69.34)	
Unmarried	372 (1.11)	145 (1.10)	227 (1.12)		343 (1.12)	13 (1.07)	
**Living areas** ^** *b* **^				0.009			<0.001
Urban	24,888 (74.37)	9,898 (75.14)	14,990 (73.86)		22,568 (73.77)	705 (58.26)	
Rural	8,579 (25.63)	3,274 (24.86)	5,305 (26.14)		8,024 (26.23)	505 (41.74)	
**Education** ^** *b* **^				<0.001			0.065
Illiteracy	20,855 (62.32)	7,075 (53.71)	13,780 (67.90)		19,056 (62.29)	722 (59.67)	
Primary school or above	12,612 (37.68)	6,097 (46.29)	6,515 (32.10)		11,536 (37.71)	488 (40.33)	
**Ethnicity** ^** *b* **^				<0.001			0.425
Han	31,373 (93.74)	12,542 (95.22)	18,831 (92.79)		28,780 (94.08)	1,145 (94.63)	
Others	2,094 (6.26)	630 (4.78)	1,464 (7.21)		1,812 (5.92)	65 (5.37)	
**Current smoker** ^** *b* **^				0.002			0.194
No	6,429 (19.21)	2,638 (20.03)	3,791 (18.68)		5,947 (19.44)	217 (17.93)	
Yes	27,038 (80.79)	10,534 (79.97)	16,504 (81.32)		24,645 (80.56)	993 (82.07)	
**Current alcohol consumer** ^** *b* **^				0.011			0.613
No	6,872 (20.53)	2,613 (19.84)	4,259 (20.99)		6,339 (20.72)	258 (21.32)	
Yes	26,595 (79.47)	10,559 (80.16)	16,036 (79.01)		24,253 (79.28)	952 (78.68)	
**Physical activity** ^** *b* **^				<0.001			0.024
No	10,166 (30.38)	4,698 (35.67)	5,468 (26.94)		9,482 (31.00)	412 (34.05)	
Yes	23,301 (69.62)	8,474 (64.33)	14,827 (73.06)		21,110 (69.00)	798 (65.95)	
**Frequency of intake fruit** ^** *b* **^				<0.001			<0.001
Almost everyday	4,401 (13.15)	2,139 (16.24)	2,262 (11.15)		4,046 (13.23)	189 (15.62)	
Quite often	6,614 (19.76)	3,160 (23.99)	3,454 (17.02)		5,919 (19.35)	147 (12.15)	
Occasionally	14,233 (42.53)	5,169 (39.24)	9,064 (44.66)		13,043 (42.64)	581 (48.02)	
Rarely or never	8,219 (24.56)	2,704 (20.53)	5,515 (27.17)		7,584 (24.79)	293 (24.21)	
**Frequency of intake vegetables** ^** *b* **^				<0.001			0.009
Almost everyday	20,441 (61.08)	8,176 (62.07)	12,265 (60.43)		18,849 (61.61)	792 (65.45)	
Quite often	8,181 (24.44)	3,347 (25.41)	4,834 (23.82)		7,304 (23.88)	238 (19.67)	
Occasionally	3,863 (11.54)	1,351 (10.26)	2,512 (12.38)		3,546 (11.59)	144 (11.90)	
Rarely or never	982 (2.93)	298 (2.26)	684 (3.37)		893 (2.92)	36 (2.98)	
**Frequency of intake meat** ^** *b* **^				<0.001			<0.001
Almost everyday	16,142 (48.23)	7,013 (53.24)	9,129 (44.98)		14,279 (46.68)	387 (31.98)	
Occasionally	11,905 (35.57)	4,168 (31.64)	7,737 (38.12)		11,215 (36.66)	584 (48.26)	
Rarely or never	5,420 (16.20)	1,991 (15.12)	3,429 (16.9)		5,098 (16.66)	239 (19.75)	
**Frequency of intake fish** ^** *b* **^				<0.001			<0.001
Almost everyday	10,916 (32.62)	5,360 (40.69)	5,556 (27.38)		9,414 (30.77)	210 (17.36)	
Occasionally	13,676 (40.86)	4,863 (36.92)	8,813 (43.42)		12,794 (41.82)	670 (55.37)	
Rarely or never	8,875 (26.52)	2,949 (22.39)	5,926 (29.2)		8,384 (27.41)	330 (27.27)	
**Self-reported health** ^** *b* **^				<0.001			0.238
Poor	4,285 (12.80)	1,514 (11.49)	2,771 (13.65)		3,932 (12.85)	173 (14.30)	
Fair	11,143 (33.30)	4,522 (34.33)	6,621 (32.62)		10,090 (32.98)	406 (33.55)	
Good	18,039 (53.90)	7,136 (54.18)	10,903 (53.72)		16,570 (54.16)	631 (52.15)	
**History of hypertension** ^** *b* **^				0.480			<0.001
No	14,478 (43.26)	5,667 (43.02)	8,811 (43.41)		13,438 (43.93)	417 (34.46)	
Yes	18,989 (56.74)	7,505 (56.98)	11,484 (56.59)		17,154 (56.07)	793 (65.54)	
**History of diabetes** ^** *b* **^				<0.001			0.905
No	32,761 (97.89)	12,772 (96.96)	19,989 (98.49)		29,950 (97.90)	1,184 (97.85)	
Yes	706 (2.11)	400 (3.04)	306 (1.51)		642 (2.10)	26 (2.15)	
**History of CVD** ^** *b* **^				0.007			<0.001
No	32,001 (95.62)	12,546 (95.25)	19,455 (95.86)				
Yes	1,466 (4.38)	626 (4.75)	840 (4.14)				
**Drinking habits** ^** *b* **^				<0.001			0.185
Boiled water	31,904 (95.33)	12,715 (96.53)	19,189 (94.55)		29,153 (95.30)	1163 (96.12)	
Un-boiled water	1,563 (4.67)	457 (3.47)	1,106 (5.45)		1,439 (4.70)	47 (3.88)	
**Drinking water sources in childhood** ^** *b* **^				<0.001			0.005
Well	21,371 (63.86)	8,472 (64.32)	12,899 (63.56)		19,691 (64.37)	726 (60.00)	
Surface water	9,974 (29.80)	3,799 (28.84)	6,175 (30.43)		8,900 (29.09)	404 (33.39)	
Spring	1,362 (4.07)	445 (3.38)	917 (4.52)		1,284 (4.20)	45 (3.72)	
Tap water	760 (2.27)	456 (3.46)	304 (1.50)		717 (2.34)	35 (2.89)	
**Drinking water sources around aged 60 years** ^** *b* **^				<0.001			<0.001
Well	18,800 (56.17)	6,650 (50.49)	12,150 (59.87)		16,940 (55.37)	591 (48.84)	
Surface water	4,246 (12.69)	1,205 (9.15)	3,041 (14.98)		3,894 (12.73)	209 (17.27)	
Spring	1,128 (3.37)	326 (2.47)	802 (3.95)		1,068 (3.49)	34 (2.81)	
Tap water	9,293 (27.77)	4,991 (37.89)	4,302 (21.20)		8,690 (28.41)	376 (31.07)	
**Drinking water sources at present** ^** *b* **^				<0.001			<0.001
Well	12,059 (36.03)	3,883 (29.48)	8,176 (40.29)		11,134 (36.40)	366 (30.25)	
Surface water	450 (1.34)	130 (0.99)	320 (1.58)		419 (1.37)	18 (1.49)	
Spring	977 (2.92)	225 (1.71)	752 (3.71)		922 (3.01)	39 (3.22)	
Tap water	19,981 (59.70)	8,934 (67.83)	11,047 (54.43)		18,117 (59.22)	787 (65.04)	

*CVD, cardiovascular diseases*.

*^a^These variables were analyzed using t-test*.

*^b^These variables were analyzed using chi-square test*.

### The Associations of Lifespan Drinking Water Sources and Habits With All-Cause Mortality

Compared to unboiled water, boiled water was not associated with all-cause mortality (*HR*: 1.060, *95% CI*: 0.995–1.129, *P* = 0.071). Compared to tap water, drinking well, surface water, and spring in childhood was not associated with all-cause mortality (*HR*: 1.044, *95% CI*: 0.931–1.171, *P* = 0.461; *HR*: 1.075, *95% CI*: 0.957–1.208, *P* = 0.225; and *HR*: 1.018, *95% CI*: 0.892–1.162, *P* = 0.791, respectively). However, drinking well (*HR*: 1.092, *95% CI*: 1.051–1.134, *P* < 0.001) and surface water (*HR*: 1.136, *95% CI*: 1.081–1.194, *P* < 0.001) around the age of 60 years, but not drinking spring (*HR*: 0.990, *95% CI*: 0.913–1.073, *P* = 0.801), was associated with all-cause mortality. Furthermore, drinking surface water at present was associated with a lower all-cause mortality (*HR*: 0.884, *95% CI*: 0.789–0.991, *P* = 0.034) (Table 2).

**Table 2:** The associations of lifespan drinking water sources and habits with all-cause mortality (*N* = 33,467).

**Factors**	**No of cases/individuals**	***HR***	**95% CI**	***P***
**Drinking habits** ^**a**^				
Un-boiled water	1,106/1,563	*Ref*		
Boiled water	19,189/31,904	1.060	0.995–1.129	0.071
**Drinking water sources in childhood** ^**b**^				
Tap water	304/760	*Ref*		
Well	12,899/21,371	1.044	0.931–1.171	0.461
Surface water	6,175/9,974	1.075	0.957–1.208	0.225
Spring	917/1,362	1.018	0.892–1.162	0.791
**Drinking water sources around aged 60 years** ^**b**^				
Tap water	4,302/9,293	*Ref*		
Well	12,150/18,800	1.092	1.051–1.134	<0.001
Surface water	3,041/4,246	1.136	1.081–1.194	<0.001
Spring	802/1,128	0.990	0.913–1.073	0.801
**Drinking water sources at present** ^**b**^				
Tap water	11,047/19,981	*Ref*		
Well	8,176/12,059	1.022	0.992–1.054	0.158
Surface water	320/450	0.884	0.789–0.991	0.034
Spring	752/977	1.011	0.936–1.092	0.784

^a^*Age, sex, weight, education levels, marital status, living areas, current smoker, current alcohol consumer, physical activity, ethnicity, dietary intake including fruits, vegetables, meat, and fish, history of hypertension, history of diabetes, history of cardiovascular disease, self-reported health, drinking water sources, and waves were adjusted*.

*^b^Age, sex, weight, education levels, marital status, living areas, current smoker, current alcohol consumer, physical activity, ethnicity, dietary intake including fruits, vegetables, meat, and fish, history of hypertension, history of diabetes, history of cardiovascular disease, self-reported health, drinking habits, and waves were adjusted*.

**Figure 2:**
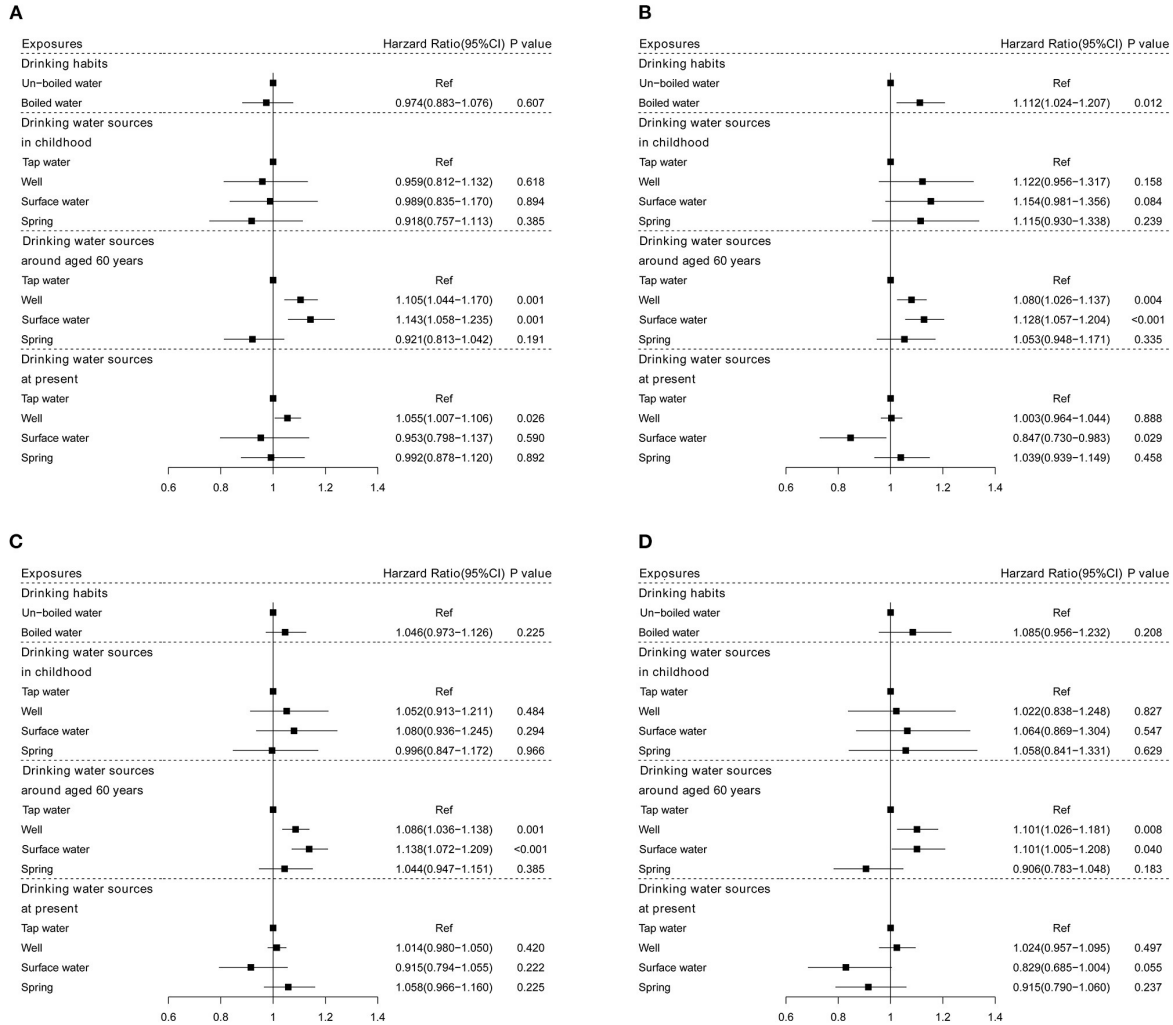
The associations of drinking water sources and habits with all-cause mortality stratified by sex and living areas. **(A)** Males; **(B)** females; **(C)** urban; and **(D)** rural.

Figure 2 shows the associations of lifespan drinking water sources and habits with all-cause mortality stratified by sex and living areas. The results stratified by sex were similar to those in the total sample. Drinking well in males and boiled water in females were associated with higher all-cause mortality (*HR*: 1.055, *95% CI*: 1.007–1.106, *P* = 0.026; *HR*: 1.112, *95% CI*: 1.024–1.207, *P*= 012, respectively). The results stratified by living areas were compared with those in the total sample.

### The Associations of Lifespan Drinking Water Sources and Habits With CVD Mortality

Table 3 shows that boiled water was not associated with CVD mortality (*HR*: 1.280, *95% CI*: 0.948–1.729, *P* < 0.107). Furthermore, drinking spring around the age of 60 years and drinking well at present were associated with a lower CVD mortality (*HR*: 0.651, *95% CI*: 0.452–0.939, *P* = 0.022; and *HR*: 0.757, *95% CI*: 0.665–0.863, *P* < 0.001, respectively).

**Table 3:** The associations of lifespan drinking water sources and habits with CVD mortality (*N* = 31,802).

**Factors**	**No of cases/individuals**	***HR***	**95% CI**	***P***
**Drinking habits** ^**a**^				
Un-boiled water	47/1,486	*Ref*		
Boiled water	1,163/30,316	1.280	0.948–1.729	0.107
**Drinking water sources in childhood** ^**b**^				
Tap water	35/752	*Ref*		
Well	726/20,417	0.759	0.534–1.079	0.125
Surface water	404/9,304	0.801	0.559–1.149	0.228
Spring	45/1,329	0.636	0.401–1.010	0.055
**Drinking water sources around aged 60 years** ^**b**^				
Tap water	376/9,066	*Ref*		
Well	591/17,531	0.872	0.755–1.007	0.062
Surface water	209/4,103	0.942	0.784–1.133	0.527
Spring	34/1,102	0.651	0.452–0.939	0.022
**Drinking water sources at present** ^**b**^				
Tap water	787/18,904	*Ref*		
Well	366/11,500	0.757	0.665–0.863	<0.001
Surface water	18/437	0.747	0.464–1.203	0.230
Spring	39/961	0.757	0.541–1.058	0.103

^a^*Age, sex, weight, education levels, marital status, living areas, current smoker, current alcohol consumer, physical activity, ethnicity, dietary intake including fruits, vegetables, meat, and fish, history of hypertension, history of diabetes, self-reported health, drinking water sources, and waves were adjusted*.

*^b^Age, sex, weight, education levels, marital status, living areas, current smoker, current alcohol consumer, physical activity, ethnicity, dietary intake including fruits, vegetables, meat, and fish, history of hypertension, history of diabetes, self-reported health, drinking habits, and waves were adjusted*.

**Figure 3:**
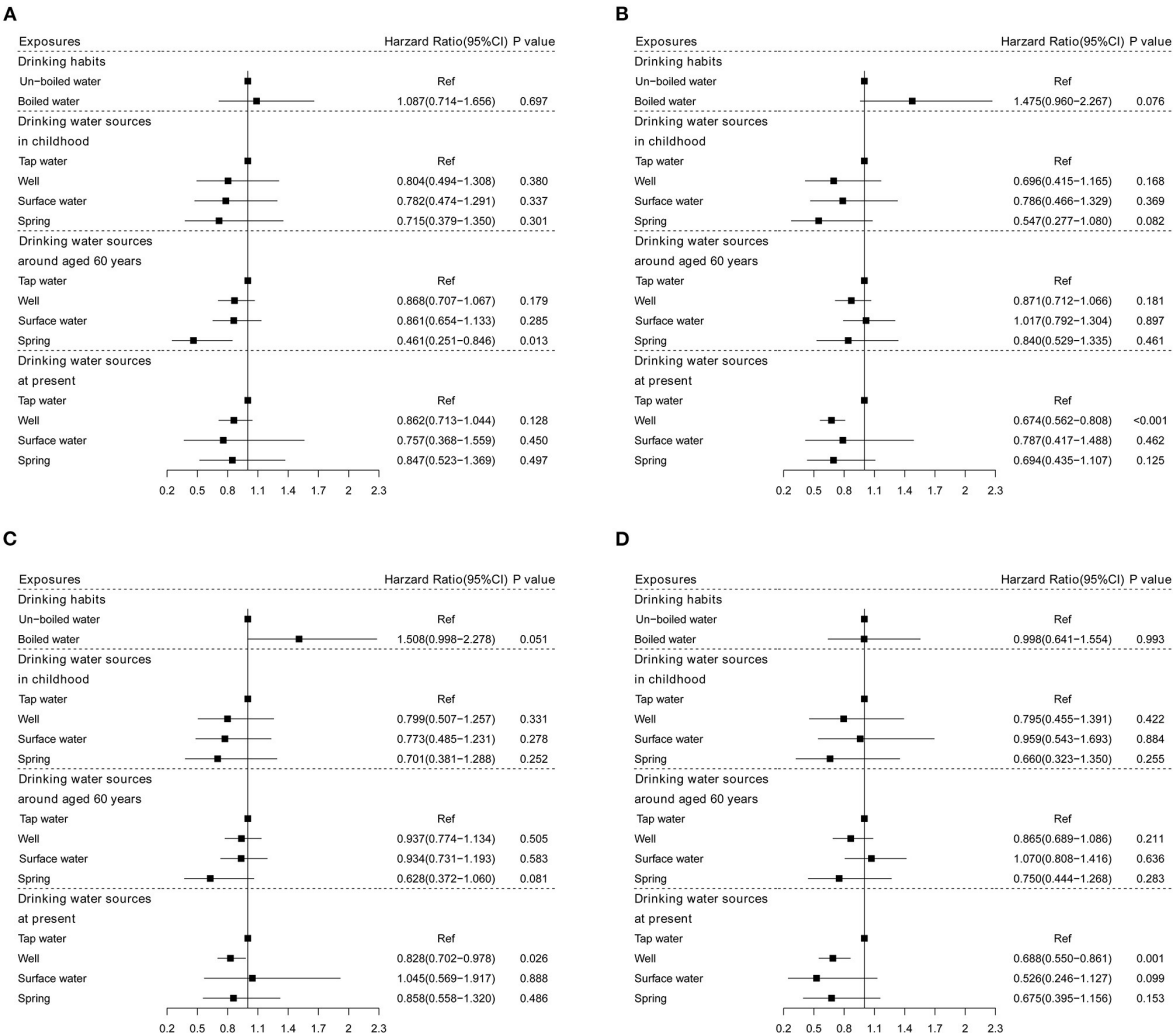
The associations of drinking water sources and habits with CVD mortality stratified by sex and living areas. **(A)** Males; **(B)** females; **(C)** urban; and **(D)** rural.

In males, there was a significant association between drinking spring water around 60-years-old and CVD mortality (*HR*: 0.461, *95% CI*: 0.251–0.846, *P* = 0.013). In females, subjects drinking well at present had a lower CVD mortality (*HR*: 0.674, *95% CI*: 0.562–0.808, *P* < 0.001) (Figures 3A,B).

When stratified by living areas, only the drinking well was associated with a lower CVD mortality in urban and rural areas (*HR*: 0.828, *95% CI*: 0.702–0.978, *P* = 0.026; *HR*: 0.688, *95% CI*: 0.550–0.861, *P* = 0.001, respectively) (Figures 3C,D).

### Sensitivity Analysis

By repeating the analysis after excluding participants dying within 2 years after baseline, Supplementary Table 1 implies that subjects drinking well and surface water around the age of 60 years and drinking well at present had higher all-cause mortality (*HR*: 1.132, *95% CI*: 1.076–1.191, *P* < 0.001; *HR*: 1.202, *95% CI*: 1.130–1.278, *P* < 0.001; and *HR*: 1.043, *95% CI*: 1.006–1.082, *P* = 0.022, respectively), which was consistent with the main results. Meanwhile, subjects drinking spring around the age of 60 years and drinking well at present had a lower CVD mortality (*HR*: 0.612, *95% CI*: 0.383–0.978, *P* = 0.040; and *HR*: 0.767, *95% CI*: 0.647–0.910, *P* = 0.002, respectively), which was comparable with the main results (Supplementary Table 2).

## Discussion

This study was designed to examine how boiled water and lifespan water sources affected CVD and all-cause mortality among older adults. The results suggested that drinking well and surface water around the age of 60 years was linked to higher all-cause mortality. Meanwhile, subjects drinking from the spring around the age of 60 years and drinking well at present had a lower CVD mortality. There was not enough evidence to support the association of boiled water with CVD and all-cause mortality.

Since there is a lack of access to clean drinking water, boiling water is the most common method for water treatment, especially in rural areas (14). The findings from a national survey revealed that over 85% of households in rural China regularly drink boiled water (12). However, a previous study found that there was no significant association between boiled water and the biomarkers of health, including eosinophilia, anemia, and obesity (15). Other studies noted that boiling water failed to reduce the risk of waterborne pathogens (16, 17). These findings supported the results of this study, which showed that drinking boiled water was not associated with mortality.

The results of this study implied that drinking well and surface water around the age of 60 years was linked to higher all-cause mortality. Well and surface water are considered unimproved water sources, while tap water is an improved water source (18, 19). It is known that surface water and groundwater are more susceptible to pathogenic microorganisms. Contaminated water is the leading cause of infectious diarrheal diseases, which directly or indirectly lead to 2.2 million deaths per year (20). It is suggested that the type of water source is a predictor of contaminated water (21). Furthermore, compared to unimproved water, an improved water source was linked to fewer microorganisms (21). A recent study indicated that the overall qualification rate of drinking water is not high, and the main challenge is still microbial pollution (1). It is documented that the major rivers and lakes in China suffer from different degrees of pollution. Approximately 9.1% of drinking water sources were not up to the quality standards of surface water or groundwater in 2018 (22). Furthermore, the treatment process of drinking water was conventional in China and failed to fully remove the harmful substances in drinking water (23, 24). On the other hand, the deficit of Ca and Mg contents in groundwater is common, which can increase oncological disease mortality (25, 26). As an endocrine disturber, the average level of the total phthalates was higher in surface water and groundwater (27). Therefore, it was rational that drinking surface water and groundwater was linked to higher all-cause mortality.

Another striking finding of this study was that drinking water from spring around 60 years of age was associated with a lower CVD mortality. The underlying mechanisms of the association of drinking water sources with CVD mortality were unknown. Tap water is commonly treated by disinfection of chlorination and fluoride. Serum chloride was considered a strong prognostic factor of heart failure (28, 29). Furthermore, chronic fluoride exposure might be associated with atherosclerosis and the cardiovascular system (30). Since long-term fluoride exposure would decrease glutathione peroxidase levels, which in turn causes systemic inflammation and endothelial activation, fluoride exposure could induce oxidative stress and promote the pathogenesis of atherosclerosis (31). Moreover, it has been documented that fluoride can accumulate in the cardiovascular system (32). Therefore, the disinfectant in tap water might be a reason why tap water was associated with a higher CVD mortality compared to spring. On the other hand, spring water is rich in minerals, such as Ca and Mg, which play a role in the prevention of CVD (33). Of course, the underlying mechanisms need to be further studied through laboratory research in the future.

## Strengths and Limitations

This study had some strengths. Firstly, this is the first study to examine the associations of boiled water and lifespan water sources with CVD and all-cause mortality in the elderly. Therefore, this study would contribute to the prevention of chronic diseases and the improvement of drinking water hygiene. Secondly, this study provided the lifespan drinking water sources for all participants. Therefore, this study provided a comprehensive picture of the associations of drinking water sources with health outcomes.

However, there were some limitations in this study. Firstly, since causes of death failed to be collected in waves 2014 and 2017–2018, the sample size of CVD mortality was not the same as that of the all-cause mortality. Secondly, public health policy in China, such as improving access to clean water and drinking water treatment, failed to be considered in this study, which might confound the observed associations. Thirdly, given that there were many elders in this study, caution should be observed when extrapolating the findings of this study to other populations. Fourthly, since information on pollutants from various resources of drinking water was not collected in the CLHLS, the underlying mechanisms of the associations of drinking water sources and habits with CVD and all-cause mortality were unknown and were not fully explained.

In conclusion, drinking water sources around 60 years of age, but not in childhood and at present, could affect mortality. Subjects drinking well and surface water around the age of 60 years had higher all-cause mortality. However, subjects drinking spring around 60 years of age and drinking well at present had a lower CVD mortality. Meanwhile, boiled water was not associated with mortality. Therefore, specific water sources and drinking habits should be proposed for populations at risk of different chronic diseases. Furthermore, more attention should be given to drinking water sources for the elderly aged around 60 years to prevent chronic diseases and increase their life expectancy.

## Data Availability Statement

The datasets presented in this study can be found in online repositories. The names of the repository/repositories and accession number(s) can be found below: https://opendata.pku.edu.cn/dataverse/CHADS.

## Ethics Statement

The studies involving human participants were reviewed and approved by the Institutional Review Board, Duke University (Pro00062871) and the Biomedical Ethics Committee, Peking University (IRB00001052–13074). The patients/participants provided their written informed consent to participate in this study.

## Author Contributions

XL contributed to writing the original draft. ZP contributed to reviewing and editing the draft. ZZ contributed to data curation and formal analysis. YZ contributed to data and results interpretation. YC contributed to the study design and conceptualization. All authors read and approved the final manuscript.

## Funding

This study was supported by the National Natural Science Foundation of China (81903416).

## Conflict of Interest

The authors declare that the research was conducted in the absence of any commercial or financial relationships that could be construed as a potential conflict of interest.

## Publisher's Note

All claims expressed in this article are solely those of the authors and do not necessarily represent those of their affiliated organizations, or those of the publisher, the editors and the reviewers. Any product that may be evaluated in this article, or claim that may be made by its manufacturer, is not guaranteed or endorsed by the publisher.
