# Supplementary material for: Associations of Boiled Water and Lifespan Water Sources With Mortality: A Cohort Study of 33,467 Older Adults
Source: Front Public Health. 2022 Jun 27;10:921738. doi: 10.3389/fpubh.2022.921738 (PMC9271665; doi:10.3389/fpubh.2022.921738)
Supplement: Supplementary file 1 [file Data_Sheet_1.pdf]

Supplementary Table 1 The associations of lifespan drinking water sources and habits with all-cause mortality removing subjects who died within the first two years after baseline (N=27,381)

Factors	*No of* cases/individuals	*HR*	*95% CI*	*P*
Drinking habits [a]				
Un-boiled water	817/1274	*Ref*		
Boiled water	13392/26107	1.058	0.983-1.140	0.133
Drinking water sources in childhood [b]				
Tap water	198/654	*Ref*		
Well	9027/17499	1.079	0.936-1.245	0.295
Surface water	4297/8096	1.115	0.965-1.288	0.140
Spring	687/1132	1.056	0.895-1.246	0.517
Drinking water sources around aged 60 years [b]				
Tap water	2970/7961	*Ref*		
Well	8456/15106	1.132	1.076-1.191	< 0.001
Surface water	2169/3374	1.202	1.130-1.278	< 0.001
Spring	614/940	0.938	0.834-1.054	0.283
Drinking water sources at present [b]				
Tap water	7567/16501	*Ref*		
Well	5821/9704	1.043	1.006-1.082	0.022
Surface water	253/383	0.902	0.794-1.026	0.116
Spring	568/793	1.006	0.920-1.100	0.898

[a] Age, sex, weight, education levels, marital status, living areas, current smoker, current alcohol consumer, physical activity, ethnicity, dietary intake including fruits, vegetables, meat, and fish, history of hypertension, history of diabetes, history of cardiovascular disease, self-

reported health, drinking water sources, and waves were adjusted.

[b] Age, sex, weight, education levels, marital status, living areas, current smoker, current alcohol consumer, physical activity, ethnicity, dietary intake including fruits, vegetables, meat, and fish, history of hypertension, history of diabetes, history of cardiovascular disease, self-reported health, drinking habits, and waves were adjusted.

Supplementary Table 2 The associations of lifespan drinking water sources and habits with CVD mortality removing subjects who died within the first two years after baseline (N=25,998)

Factors	*No of* cases/individuals	*HR*	*95% CI*	*P*
Drinking habits [a]				
Un-boiled water	27/1212	*Ref*		
Boiled water	668/24786	1.320	0.889-1.962	0.169
Drinking water sources in childhood [b]				
Tap water	16/647	*Ref*		
Well	428/16694	0.882	0.530-1.466	0.628
Surface water	226/7553	0.891	0.531-1.495	0.663
Spring	25/1104	0.647	0.338-1.241	0.190
Drinking water sources around aged 60 years [b]				
Tap water	208/7754	*Ref*		
Well	347/14054	0.892	0.736-1.080	0.242
Surface water	119/3272	0.889	0.697-1.133	0.342
Spring	21/918	0.612	0.383-0.978	0.040
Drinking water sources at present [b]				
Tap water	440/15599	*Ref*		
Well	219/9246	0.767	0.647-0.910	0.002
Surface water	10/371	0.605	0.319-1.145	0.122
Spring	26/782	0.790	0.520-1.202	0.271

[a] Age, sex, weight, education levels, marital status, living areas, current smoker, current alcohol consumer, physical activity, ethnicity, dietary intake including fruits, vegetables, meat, and fish, history of hypertension, history of diabetes, history of cardiovascular disease, self-

reported health, drinking water sources, and waves were adjusted.

[b] Age, sex, weight, education levels, marital status, living areas, current smoker, current alcohol consumer, physical activity, ethnicity, dietary intake including fruits, vegetables, meat, and fish, history of hypertension, history of diabetes, history of cardiovascular disease, self-reported health, drinking habits, and waves were adjusted.
